# A dynamic stimulus distribution methodology for efficient large-scale pairwise relational data collection

**DOI:** 10.1016/j.mex.2026.103839

**Published:** 2026-02-20

**Authors:** Zhang Chuyin, Lin Lipeng, Naotsugu Tsuchiya

**Affiliations:** a School of Psychological Sciences, Faculty of Medicine, Nursing & Health Sciences, Monash University, Melbourne, Victoria, Australia; b Turner Institute for Brain and Mental Health & School of Psychological Sciences, Faculty of Medicine, Nursing, and Health Sciences, Monash University, Melbourne, Victoria, Australia; c Center for Information and Neural Networks (CiNet), National Institute of Information and Communications Technology (NICT), Suita, Osaka 565-0871, Japan; d Advanced Telecommunications Research Computational Neuroscience Laboratories, 2-2-2 Hikaridai, Seika-cho, Soraku-gun, Kyoto 619-0288, Japan; e Theoretical Sciences Visiting Program (TSVP), Okinawa Institute of Science and Technology Graduate University, Onna, 904-0495, Japan

**Keywords:** Psychophysics, Online experiment, JavaScript, PsychoPy, Pavlovia, Pairwise relationship, Similarity matrix, Balanced number of observations, Stimulus selection

## Abstract

Subjective comparisons of aspects of experience provides reliable and powerful numerical data, which can provide us means to characterize structures of consciousness. Yet, an exhaustive set of comparative and pairwise judgements among N stimuli requires N^2^ trials, which is costly for in-lab face-to-face data collection from a participant. By utilizing an online experimental platform, it is easy to recruit many participants, randomly distributing a small proportion of all possible pairs. However, random assignment is not efficient in obtaining data uniformly across all pairs of stimuli. Here, we introduce a new method for minimizing variance in trial counts across stimulus pairs, by integrating PsychoPy with GitHub Gist, which records the frequency with which each pair has been presented. We provide JavaScript code that can be incorporated into customized code chunks in PsychoPy. The program can be run on Pavlovia for online participants, and we show the effectiveness of our method.

• The frequencies that each stimulus pair has been shown are stored on GitHub Gist.

• When a new participant starts on Pavlovia, our methodology reads the frequencies, selects the least presented stimuli for the participant, and updates the frequencies.

• The frequencies get dynamically balanced for efficient data collection.

## Graphical abstract

**Figure:**
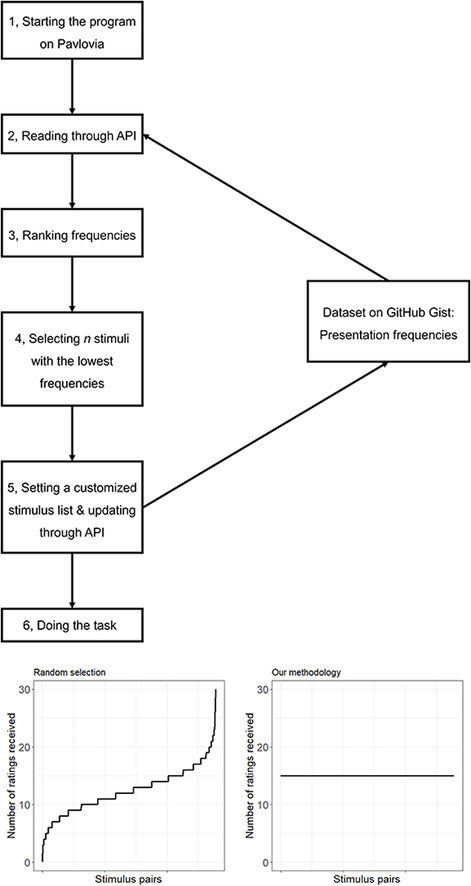

## Specifications table

**Table:** 

**Subject area**	Psychology, consciousness
**More specific subject area**	Psychophysics, online experiment
**Name of your method**	Dynamic balancing of stimulus presentation on Pavlovia
**Name and reference of original method**	NA
**Resource availability**	https://doi.org/10.17605/OSF.IO/GS87V

## Background

Characterization of relations among mental relata has a long history in psychology [[1], [2], [3], [4]]. Recently, a relational approach has been proposed as a promising way to characterize qualitative aspects of experience (or qualia; e.g., [[5], [6], [7],^8^]). Pairwise judgement is one of many approaches to quantify the relations (other approaches include e.g., odd-one-out [9,10], sorting [11], and arrangement task [12]). Pairwise presentations of stimuli (either simultaneously or in a temporal sequence; [13]) allows several advantages in experimental manipulations that are difficult to attain with other methods and that are often critical in characterization of qualia. Most important is its precise control of the duration and the location of stimulus presentation. For example, under the pairwise brief presentation, color qualia similarity structures can be compared between fovea and periphery [14], which is impossible with other methods. Similarly, manipulation of attention [15] and stimulus visibility [16] are also difficult to attain with the other methods. However, gathering enough data to accurately characterize these relations is time-consuming and resource-intensive. We will first clarify what constitutes "sufficiency" in this context, then propose a methodology to help researchers collect sufficient data more efficiently.

Sufficiency of relational data has two meanings. First, comprehensively characterizing relations among relata requires completeness: for N relata, researchers need responses for all N(N-1)/2 unique pairs, at least. This assumes that 1) they do not need to measure N pairs of two same stimuli (assuming participants will reliably rate them to be most similar always) and that 2) they can assume the symmetry of relational judgements. Note however, both assumptions are often empirically violated [[4], [13], [15], [16]]. Second, for a given pair of stimuli, the estimate of the mean judgment across participants will become more accurate as the number of repetition increases. Thus, for sufficient data collection, it would be ideal to collect multiple estimates across the full relation matrix.

To collect data in the full relation matrix, researchers can ask individual participants to rate all combinations, creating individual-level relationship matrices. This works well for small stimulus sets. For example, if we have 10 images, that provides 45 pairs. Assuming each trial takes 30 seconds, it is feasible to let each participant rate the similarity of all the unique pairs. But it becomes time-consuming and burdensome when the set size increases (although it is not impossible, e.g., [[17], [18]]). Alternatively, as we pursue in this paper, they can distribute the task across multiple participants using an online experimental platform (e.g., Prolific, etc), where each participant rates only a subset of stimulus combinations. Aggregating the data to create a group-level matrix (e.g., [5,6]) is easy through recruitment of a huge number of online participants. The key is to appropriately distribute stimulus subsets to cover all pairwise relations for large stimulus sets. Random distribution of stimulus subsets is inefficient. Randomness causes each pair's presentation frequency to be skewed.

Our novel algorithm dramatically solves this problem as shown below.

## Method details

We present a dynamic stimulus allocation method that adjusts each participant's stimulus set based on real-time data stored in the cloud. Our method minimizes inter-pair variance in trial counts. This helps achieve the required number of observations with the minimum sample size. Our method is based on a common online experiment software, PsychoPy, which can be run on an online platform, Pavlovia. We insert JavaScript code into customized code chunks, which can be easily adapted for any experiment that is directly written in JavaScript. Future researchers might also adapt it for different experimental designs (we will introduce an example later).

### Motivations

In our recent study [6], to characterize the experience of emotions, we asked participants to rate the similarity between a pair of emotion videos, presented in a temporal sequence. Upon seeing the pair, participants rated the similarity between them. As we had a stimulus set of 75 videos, we needed to test 75×74÷2=2775 pairs of videos, removing comparisons of the same videos and disregarding the order effects. To keep the duration of the experiment per participant reasonably short (∼30 min), we limited the number of trials per participant to 75 (+ 50 repetitions of the same pairs to assure the reliability of participants’ responses and questionnaire, relevant for our experiment in particular).

**Fig. 1:**
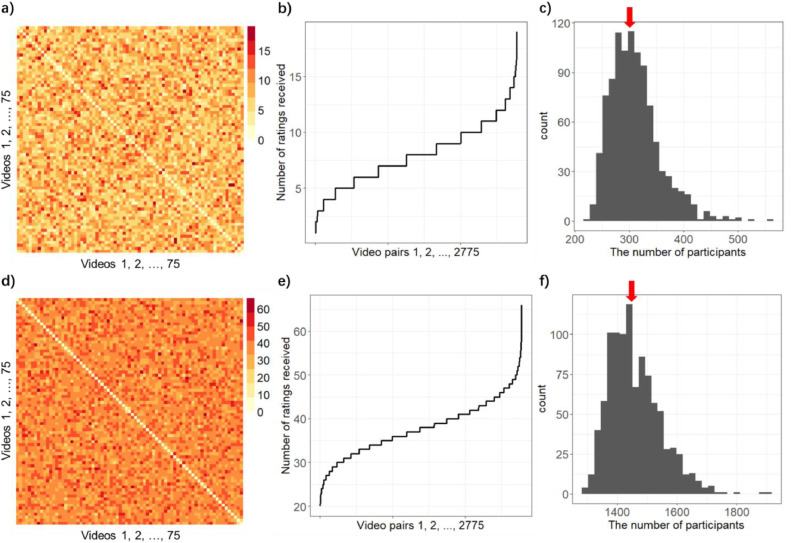
**Simulated outcomes for random selection.** a) The response frequency matrix from one example simulation in which every pair of videos has at least one rating. X and Y axes are video ids. Each cell c(i, j) of a matrix represents the number of responses for a pair of videos i (column) and j (row), presented first i then j. For this analysis, we have already symmetrized the matrix, such that Sc(i, j) = c(i, j) + c(j, i). The number is colorcoded. b) The number of responses (y-axis) is shown for each video pair, with video pair IDs on the x-axis sorted in ascending order by response count. c) The distribution of the minimal number of participants (x-axis) required to achieve at least 1 rating for each video pair. Over 1000 times of simulation (each simulation resulting in a Fig. like a and b), we needed ∼304 (median, red arrow) participants. a) and b) is coming from an exemplar simulation, which achieved the at least 1 rating for each video pair at 304 participants. d) & e) One example simulation in which every pair of videos has at least 20 ratings. The format is the same as a) & b). f) The distribution of the minimal number of participants required to achieve at least 20 ratings for each video pair. The format is the same as c) (1000 times repetitions. Median = 1444, red arrow). The number of participants we needed for d) and e) is marked by the red arrow.

What is the best way to assign a sequence of 75 videos to each participants so that all 2775 pairs are evenly covered? One way is to randomly select them, which we can program relatively easily. Fig. 1 shows some simulated outcomes this way. For example, when this random assignment scheme achieves at least one rating per pair, some video pairs have been already selected more than 15 times (as shown in Figs. 1a and 1b). If we repeat such random selection 1000 times, the distribution of the minimally required number of participants is shown in Fig. 1c. The median of the 1000 simulations is 304, which means if we want each video pair to be rated more than once, we usually need more than 300 participants. If a researcher targets at least 20 ratings for each pair, the number of total participants that is required increases to ∼1400 participants (shown in Figs. 1d-f).

Therefore, we aim for each pair to be judged by equal numbers of participants. To achieve that, our novel methodology dynamically selects the pairs that have been presented least, with two additional advantages. First, our methodology allows researchers to customize the format or features of their stimulus list. Second, for massive data collection online, researchers often recruit many participants in parallel. Our methodology works well in such a situation (see ***Technical step 4*** for details).

Now we will introduce the technical details step by step.

#### Technical step 1: upload your frequency onto GitHub Gist

**Fig. 2:**
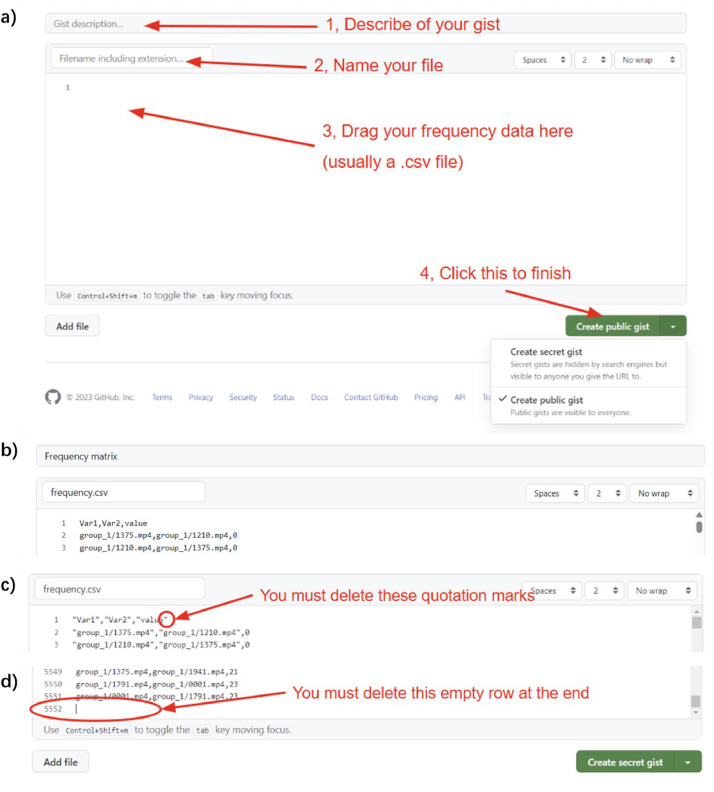
**To upload your frequency data onto GitHub** G**ist.** a) The empty page at GitHub Gist where you can upload your current frequency. b) Our melted frequency matrix. c) You must delete these quotation marks. d) You must delete the empty row at the end.

Please go to gist.github.com and log in your GitHub account. Then you will be directed to a page where you can create a new gist (Fig. 2a). You should already have data for the current trial counts on your computer, in the format of a melted matrix (Fig. 2b), and written in a .csv file. This format can be generated by applying the melt() function to a matrix in R. If you start from scratch, begin with a melted matrix where all values equal 0; if you have existing data, upload your current response frequency matrix to use our methodology for continued data collection. You need to ensure there are no quotation marks (Fig. 2c) and you delete the empty row at the end (Fig. 2d).

In our example (Fig. 2b), the first column contains the file paths (a relative path that the PsychoPy program can read) of one video, and the second column contains the file paths of the other video. The third column contains how many times each pair of videos has been shown after recruiting the first 457 participants. Note here we do not consider the order for this example. If you want to study the order effects, please record (stimulus A, stimlus B) and (stimlus B, stimlus A) separately.

After uploading your frequency in the correct format, you can create the gist. You need to select the “Create public gist” option so that all participants’ browsers can access the file.

#### Technical step 2: PsychoPy program reads the frequency from GitHub Gist

**Fig. 3:**
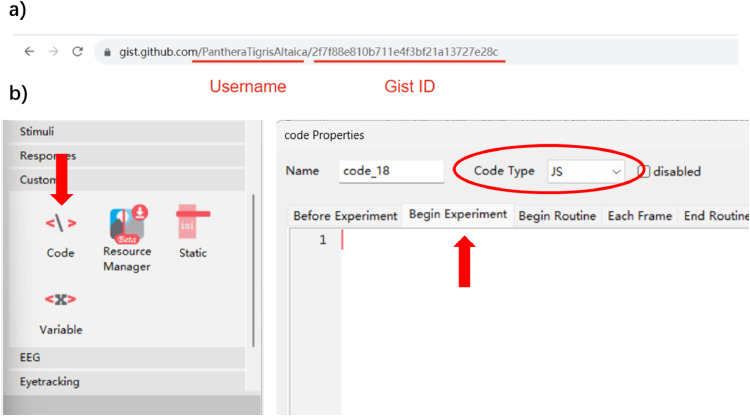
**Demonstration for step 2.** a) How to find the ID of your gist. This will be used later in the code. b) How to insert the code for reading the frequency.

The next step is to make your experiment program able to read the frequency from GitHub Gist. After creating your gist, you should be directed to the page of the gist. In your browser, you can find the link to it. There are two strings after “gist.github.com” (Fig. 3a). The first one is your username. The second one is the ID of your gist. We will use the ID for reading.

Let’s open your experiment program on PsychoPy and insert the code. You can find the code component under “Custom” (Fig. 3b). You can insert your code into your first routine. On the code properties page (Fig. 3b), please ensure the code is under “Begin experiment” and the code type is JavaScript. In the following steps (2-4), **the code in bold text** represents things you need to replace with information from your own project. And we recommend putting all the code under the same component.

Now we will show you the code you should insert. First, we use the .ajax() from jQuery to access the frequency data on GitHub Gist. Here is the code:

*//read the frequency data*

*// Step 1: Fetch Gist Details*

*$.ajax({*

*url: '* https://api.github.com/gists/ ***2f7f88e810b711e4f3bf21a13727e28c*** *', // Replace with url of your gist*

*type: 'GET',*

*async: false,*

*success: function(gistData) {*

  *// Step 2: Extract the Raw URL for the CSV file*

  *var csvFileName = '****frequency.csv****'; // Replace with your CSV file's name*

  *var rawUrl = gistData.files[csvFileName].raw_url;*

  *// Step 3: Fetch the CSV Data*

  *$.ajax({*

  *url: rawUrl,*

  *type: 'GET',*

  *dataType: 'text',*

  *async: false,*

  *}.done(successFunction_v);*

*},*

*error: function(error) {*

  *console.error('Error fetching Gist details:', error);*

*}*

*});*

When adapting this code, you should put the ID of your gist after “https://api.github.com/gists/” as the url, and define the csvFileName with your own file name. This code will read all the frequency data in Fig. 2b as a string. After that, the code will execute a function called “successFunction_v” to process the data and transform it into a suitable format. Here is the function:

*//pre-process the long string*

*var frequency1 = Array();*

*function successFunction_v(data) {*

*var allRows = data.split('\n'); // split rows at new line*

*for (var i = 1; i < allRows.length; i++) {*

  *var currentLine = allRows[i];*

  *var temp = currentLine.split(',');*

  *var current = Array();*

  *current.push(temp[0]);*

  *current.push(temp[1]);*

  *current.push(Number(temp[2]));*

  *frequency1.push(current);*

*}*

*}*

We create a global 2-D array (frequency1) to store the processed data. When adapting this code, you should adjust it based on the format of your own frequency data. Here we ignore the titles of columns (by “i = 1”), but we will add them soon by converting the data into an object array:

*//convert to an object array*

*keys = [* ***'video1′, 'video2′, 'value'*** *]; // Replace with your column names*

*arrayChangeJson = (source, keys) => source.map(r => keys.reduce((total, k, i) => {*

*total[k] = r[i]*

*return total*

*},{}))*

*let frequency = arrayChangeJson(frequency1, keys);*

*util.shuffle(frequency);*

*frequency.sort((a, b) => a.value - b.value);*

At the end, we shuffle the stimuli and rank them according to their frequency. The purpose of shuffling is to randomize the order of stimuli with the same frequency.

#### Technical step 3: generate a stimulus list for the current participant

In this step, you need to generate a stimulus list based on the frequency data and your experimental design. Your program will present the stimulus to the current participant as per your list. We cannot tell you how to do it as this is a highly customized part. (And after getting the list, you need to utilize it in your own stimulus-presentation routines in PsychoPy.) For example, you might want to restrict each video to be shown no more than 3 times, or introduce a double-pass design (i.e., show the same stimulus pairs twice to a participant, for a test-retest reliability check; see [5,6]).

As an example for this paper, we show the videos one by one, so our stimulus list is a sequence of videos. We first find the start point:

*//start point*

*lowest1 = frequency[0].value;*

*let lowest = frequency.filter(videos => videos.value === lowest1);*

*start = lowest[0];*

***sequence*** *= [****start.video1****,* ***start.video2****]; // customize your stimulus list*

Then we extend the sequence (stored in “sequence”) according to the rank of frequency. For details of our experiment, please see our paper [6].

#### Technical step 4: update the frequency matrix onto GitHub Gist

We update the frequency data right after generating the stimulus list and before starting the experiment. There are two advantages to doing so.

First, we can keep the stimuli shown to all participants, including those who do not complete the experiment. Sometimes, researchers might be interested in the relationship between stimuli and drop-out rate. Second, we can adjust the frequency matrix per participant optimally in cases where the actual frequency matrix is changing dynamically. The frequency matrix, when read to generate the stimulus list, must reflect the current frequency of all stimulus combinations at that moment. We believe this situation is very common in the types of psychological experiments for which this method is intended. In such experiments, researchers typically collect many relational data points from each participant, with sessions often lasting 30–60 minutes. To leverage online testing, participants are usually recruited in large numbers simultaneously. If frequency data were updated only after each participant completes the entire experiment, many stimulus lists would be generated from identical, outdated frequency information, reducing the efficiency of our algorithm. In contrast, updating after each trial would be impractically frequent. Our current method strikes a balance between controlling the overall structure of the stimulus list and maintaining efficient, near–real-time updates to the frequency data. (as described in step 3).

To prepare for the new frequency matrix, you need to update it in your PsychoPy program, and then convert it into a long string (i.e., the same as the raw data you read from GitHub Gist). Before updating, we highly recommend you to read the frequency data again, using the same code in step 2, to ensure you are modifying the most up-to-date data. In our case, the newly read frequency data is stored in an object array called “currentfrequency”. Here is the code:

*//update the frequency*

*for(var i = 1; i < **sequence**.length - 49; i++){*

*var v1 = **sequence**[i-1];*

*var v2 = **sequence**[i];*

*currentfrequency.forEach(row => {*

  *if (row.**video1** === v1 && row.**video2** === v2) { // Replace with your column names*

  *row.****value*** *+= 1;*

  *} else if (row.****video1*** *=== v2 && row.****video2*** *=== v1) { // Replace with your column names*

  *row.****value*** *+= 1;*

  *} //Here the order is not considered. If you want to consider the order, delete the else if part*

  *});*

*}*

*var newfrequency = Array();*

*newfrequency.push[* ***'Var1′,'Var2′,'value'*** *]; //Replace with the format of your .csv file*

*for(var i = 0; i < currentfrequency.length; i++){*

*var temp = currentfrequency[i];*

*var tempvalue = Object.values(temp);*

*newfrequency.push(tempvalue);*

*}*

*for(var i = 0; i < newfrequency.length; i++){*

*temp = newfrequency[i];*

*newfrequency[i] = temp.join();*

*}*

*filecontent = newfrequency.join('\n');*

In this code, we first update the frequency, and then convert the object array into a 2-D array called “newfrequency”. After that, we connect the rows together and store the results in a string called “filecontent”, which we will use to overwrite our gist.

**Fig. 4:**
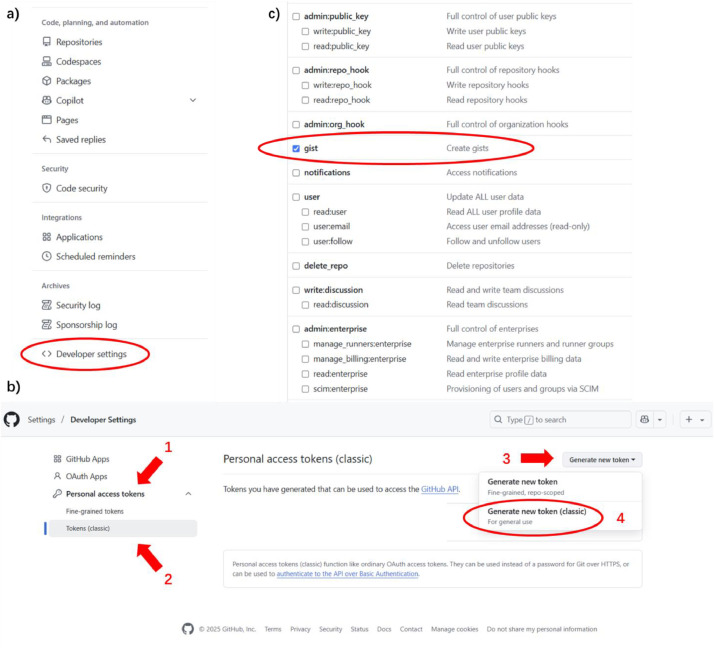
**To generate a personal access token on Git**H**ub.** a) Go to your account settings and find the developer settings. b) How to generate a token. The numbers mean the order of clicks. c) Remember to tick “gist” under “Scopes” so your token can be used to modify your gists.

Before your PsychoPy program can modify your gist, an authorization process is required by GitHub. First, you need to obtain a personal access token from GitHub. You can go to your GitHub settings. On the left side, there is a “Developer settings” at the end (Fig. 4a). Then, on the left side, you click “Personal access tokens” and “Tokens (classic)”. After that, you can click the “Generate new token” at the top-right corner and generate a classic token (Fig. 4b). On the new page, you can name your token, set an expiration date, and define what this token can do (called “scopes”). Remember to tick “gist” to allow your token to be used for gists (Fig. 4c). After everything is settled you can click the “Generate token” at the bottom and obtain your token.

We use the .ajax() again to overwrite our gist with the long string that stores the updated frequency data (“filecontent”). Here is the code:

*//overwrite*

*var githubToken =* ***'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′****; // Replace with your GitHub token*

*var fileName =* ***'frequency.csv'****; // Replace with your .csv file name*

*var gistId =* ***'2f7f88e810b711e4f3bf21a13727e28c'****; // Replace with the ID of your gist*

*$.ajax({*

*url: '* https://api.github.com/gists/ *${gistId}',*

*type: 'PATCH',*

*beforeSend: function (xhr) {*

  *xhr.setRequestHeader('Authorization', 'token ' + githubToken);*

*},*

*data: JSON.stringify({*

  *files: {*

   *[fileName]: {*

    *content: filecontent*

   *}*

  *}*

*}),*

*success: function (response) {*

  *console.log('Gist updated successfully.', response);*

*},*

*error: function (error) {*

  *console.error('Error updating Gist:', error);*

*}*

*});*

In addition to the code for reading in step 2, we use the personal access token we just generated for authorization. You need to define the “githubToken” with your own token.

These are all the technical steps we implement. As participants sign in and do the task, the program can dynamically generate stimulus lists for each person to show the stimuli evenly, and keep updating the frequency data. There are two issues you need to notice while using our methodology. Please see the Limitations section below.

## Method validation

**Fig. 5:**
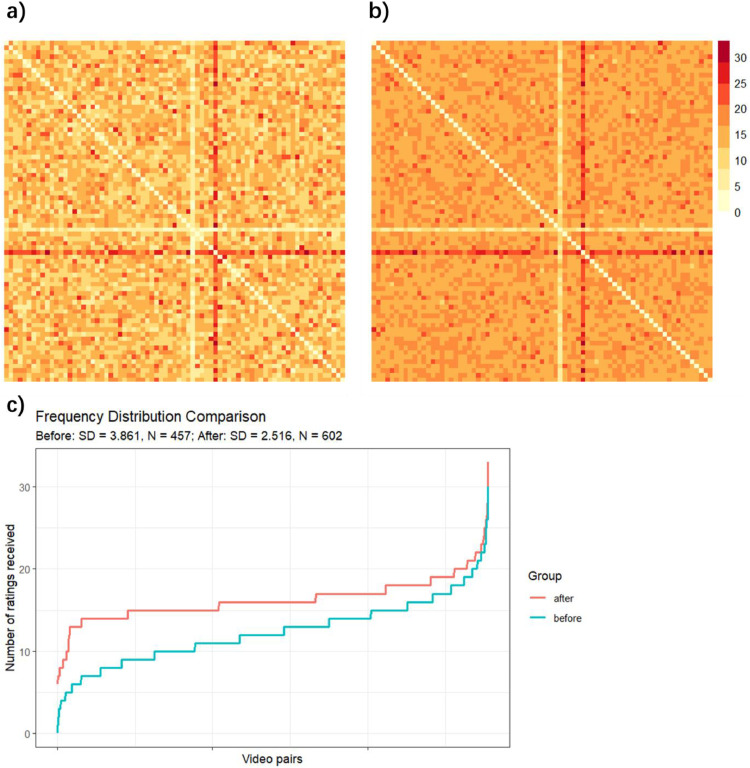
**Response frequency of video pair presentations after implementing the methodology for 145 additional participants.** a & b) The frequency matrices where each cell represents a pair of videos. Redder color means the pair of videos is shown more frequently. Compared with a) (before implementing the methodology), the color in b) (after implementing the methodology) becomes more uniform. c) The distribution of observation frequencies before (blue) and after (red) implementing the methodology. X axis contains every video pair and Y axis is the number of ratings received. Our methodology biases the less-presented video pairs and makes the left-hand tail more uniform.

The real frequency data from Lipeng et al. [6] validates our methodology. In the study, we first ran a randomization selection strategy for N = 457 participants (shown in Fig. 5a). Note that we had a bug in the code, which repeated one video more frequently (the darker cross) and the other one stimulus less (the lighter cross).

After that, we noticed the issue of unbalanced stimulus selection. We fixed the bug and developed the current methodology. After implementing the current methodology, we recruit another group of 145 participants. The frequency results of the total 602 participants are shown in Fig. 5b, which tends to be more even compared with Fig. 5a.

Fig. 5c shows the distributions of presentation frequencies across 2775 video pairs, for before and after implementing the methodology. As we see, before the implementation, 1 video pair has not been shown to participants at all, and 94 video pairs were shown no more than 5 times. But after the implementation, all video pairs were shown more than 6 times and 98% of the video pairs were shown more than 10 times. A Levene’s test shows a significant decrease in the variance of frequency after implementing the current methodology, *F*(1, 5548) = 510.17, *p* < .001. That means the video pairs are shown more evenly after implementing the current methodology. And more importantly, our methodology can guarantee that no stimuli are shown much less frequently. Thus, our methodology can help effectively collect data for every stimulus pair.

### Significance of our methodology

While some online experiment platforms offer a solution to the problem in this paper, their solution appears in a black box. For example, Meadows (https://docs.meadows-research.com/stimuli/selection/, accessed on 2nd Dec. 2025) offers something similar to what we offer. But they say “this is a somewhat complicated algorithm so please contact us with any questions”. Through our methodology, users know what their program is actually doing and can modify and control it for any way they want.

Recently, Pavlovia offered a built-in function called Shelf which can achieve similar goals. The server stores a dictionary which the experiment program can interact with through Shelf. An obvious advantage of Shelf is that the data is stored on Pavlovia so it can be easily accessed, but the data structure is not straightforward at the moment. According to the introduction by Pavlovia (see https://www.psychopy.org/online/shelf.html, accessed on 2nd Dec. 2025, for technical details), Shelf works well for a situation where the number of participants is settled, and the stimulus list for each participant is pre-generated. E.g., the researcher aims to recruit 100 participants and generates 100 stimulus lists which balance the stimulus selection. Then Shelf can ensure each stimulus list is used only once. Our methodology works beyond this situation and offers more flexibility and adaptability as the research goes. For example, our methodology also works well for a situation where researchers do not plan an exact sample size, employing a more sophisticated and efficient Bayesian stopping rule (e.g., recruiting participants until the Bayes Factor reaches 10 or 1/10 [19,20].

### Existing adaptations of our methodology

There are some studies that have already adapted the current methodology. For example, Watanabe et al. [21] used it to balance the selection of 93 color patches.

## Limitations

When using our method, two issues should be noted. They do not affect the core functionality, but we outline them below along with solutions.

***Issue 1: when two participants starting at the exactly same time, one might be lost***

**Fig. 6:**
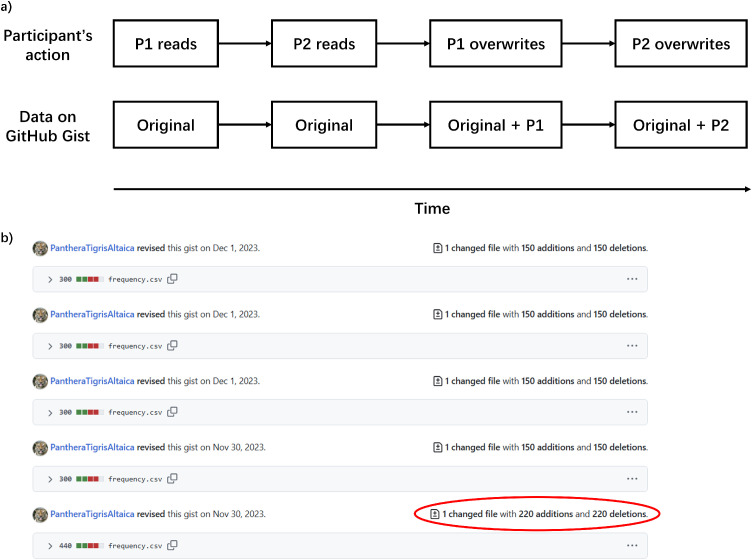
**Multiple participants might start at the same time.** a) The timeline when this makes an influence. Two participants (P1 & P2) read the same frequency data from GitHub Gist. P2’s updates overwrite P1’s. As a result, the frequency updates from P1 are lost. b) We can notice the issue in the revisions of our gist. In our case, each participant should change 150 frequencies (75 video pairs, without considering the order). An unusual number (i.e., 220 here) means some information is overwritten.

Although very quick, there is a time gap (usually < 0.1 s) between reading our gist and overwriting it. After one participant reads the frequency data, and before this person updates, if another participant starts the experiment and reads the data, the updates from the first participant will be lost (see Fig. 6a).

In practice, when an advertisement is published on Prolific, a large number of participants can sign up immediately. Under such a situation, this issue can arise, but it is notable in the revisions of the gist as an unusual number of changes (see Fig. 6b for our example).

***Issue 2: drop-out participants are still recorded***

Since the program updates the frequency at the beginning of the experiment, even though a participant drops out before completion, their frequency data is still updated. That means the frequency of actual data we collect might be different from what our gist shows.

### Work-around

At the moment, we do not have a perfect solution to these two issues. As a work-around to Issue 1, we recommend recruiting participants in several batches, not all at once. For example, if you aim to recruit 1,000 participants, you can open 50 slots first. Then, you add 50 more slots every 10 or 20 minutes. That will reduce the density of participants coming in and also give you some time to check the gist revisions to notice the issue.

As a work-around to Issue 2, we recommend to update the gist regularly with the frequency from actual data. If participants quit the task before the completion, Pavlovia would mark them, so you can easily remove them from your data storage. By analyzing the trial counts from the actual completed participants, you can alleviate this problem.

## Ethics statements

We obtained ethical approval for the online psychological studies that utilises current methodology (i.e., [6]) from the Monash University Human Research Ethics Committee (Project ID: 41190).

## CRediT author statement

Zhang Chuyin: Conceptualization, Methodology, Software, formal analysis, resources, writing - original draft, writing - review & editing, visualization, project administration.

Lin Lipeng: Software, formal analysis, investigation, resources, data curation, writing - review & editing, visualization.

Naotsugu Tsuchiya: Conceptualization, writing - review & editing, supervision, funding acquisition.

## Supplementary material *and/or* additional information [OPTIONAL]

The code for analysis and generating the Fig.s can be found at https://doi.org/10.17605/OSF.IO/GS87V

## For a published article

NA

## Declaration of interests

The authors declare that they have no known competing financial interests or personal relationships that could have appeared to influence the work reported in this paper.

## Data Availability

I have shared the link to my data and code at the Attach File step.
